# Healthcare professionals’ longitudinal perceptions of group phenomena as determinants of self-assessed learning in organizational communities of practice

**DOI:** 10.1186/s12909-022-03137-9

**Published:** 2022-02-03

**Authors:** François Durand, Lucie Richard, Nicole Beaudet, Laurence Fortin-Pellerin, Anahi Morales Hudon, Marie-Claude Tremblay

**Affiliations:** 1 Montfort Research Chair in Organization of Health Services, Telfer School of Management, University of Ottawa, 55 Laurier Avenue East, Ottawa, Ontario K1N 6N5 Canada; 2 Faculté des sciences infirmières, Université de Montréal, Montréal, Canada; 3 Direction de santé publique de Montréal, Montréal, Canada; 4 Institut de recherche en santé publique, Université de Montréal, Montréal, Canada; 5 Faculty of Human Sciences, Saint Paul University, Ottawa, Canada; 6 Département de médecine de famille et de médecine d’urgence, Université Laval, Quebec, Canada

**Keywords:** Collaboration, Psychological safety, Commitment, Learning, Trust, Group processes, Learning, Longitudinal study, Perception

## Abstract

**Background:**

Given the importance of continuous learning as a response to the increasing complexity of health care practice, there is a need to better understand what makes communities of practice in health effective at fostering learning. Despite the conceptual stance that communities of practice facilitate individual learning, the scientific literature does not offer much evidence for this. Known factors associated with the effectiveness of communities of practice – such as collaboration, psychological safety within the community, and commitment to the community – have been studied in cross-sectional qualitative designs. However, no studies to date have used a quantitative predictive design. The objective of this study is to assess how members of a community of practice perceive interactions among themselves and determine the extent to which these interactions predict self-assessed learning over time.

**Methods:**

Data was collected using validated questionnaires from six communities of practice (*N* = 83) in four waves of measures over the course of 36 months and was analysed by means of General Estimating Equations. This allowed to build a longitudinal model of the associations between perceptions of collaboration, psychological safety within the community, commitment to the community and self-assessed learning over time.

**Results:**

Perception of collaboration in the community of practice, a personal sense of psychological safety and a commitment to the community of practice are predictors longitudinally associated with self-assessed learning.

**Conclusions:**

In terms of theory, conceptual links can be made between intensity of collaboration and learning over time in the context of a community of practice. Recent work on psychological safety suggests that it is still unclear whether psychological safety acts as a direct enhancer of learning or as a remover of barriers to learning. This study’s longitudinal results suggest that psychological safety may enhance how and to what extent professionals feel they learn over time. Commitment towards the community of practice is a strong predictor of learning over time, which hints at differential effects of affective, normative and continuance commitment. Communities of practice can therefore apply these findings by making collaboration, psychological safety, commitment and learning regular reflexive topics of discussion.

## Background

Communities of practice are social systems composed of individuals who share problems or are passionate about a topic, and who extend their understanding and proficiency by interacting on an continuing basis [1]. They are “‘tightly knit’ groups that have been practicing together long enough to develop into a cohesive community with relationships of mutuality and shared understandings.” [2] Learning is the cornerstone of communities of practice for novices and more experienced professionals alike [3]. Edmondson [4] explains that learning in a collective, such as a team or a community of practice, is determined by interpersonal perceptions that are affected by individual members’ perceptions of the social climate. How an individual community member perceives and reflects on what happens in their community of practice is therefore important. Healthcare professionals are asked to collaborate interprofessionally (i.e., across background and expertise silos) as a response to the increased of health needs and the delivery of patient-centred care and services [5, 6]. Interactions in a group influence its members [7], impacting their psychological characteristics such as cognition, motivation and affect; in turn, individual members’ cognition, motivation and affect influence the group as a whole [8]. In a community of practice, reflexivity is important in terms of how its members monitor how the community works, with the goal of improving learning.

Healthcare offers many examples of applications of communities of practice. These include communities of practice established to: integrate knowledge on innovativeness in prostate cancer treatment [9]; understand how policies on services for elderly patients came to be [10]; help less advanced projects in integrated child health information systems learn from more advanced ones [11]; evaluate the effectiveness of the online environment for knowledge exchange among rural and urban emergency clinicians [12]; implement an infrastructure and provide tools to improve cancer surgery practice [13]; increase physicians’ implementation of evidence-based care [14]; improve collaboration and knowledge dissemination regarding cancer control [15]; evaluate knowledge translation leading to improved tobacco-control policies and practices [16]; contribute to learning in a global healthcare environment [17]; improve palliative care knowledge and self-efficacy among interprofessional health care providers [18]; and foster quality assurance in healthcare assessment [19].

A focus on what fosters learning in community of practices is important because, despite the conceptual stance that communities of practice provide a context that facilitates individual learning [1], the scientific literature does not offer much evidence for this, particularly for communities of practice in the early stages of development [20]. In fact, healthcare organizations wishing to capitalize on communities of practice in order to achieve organizational goals need to better understand the factors that predict community of practice’s outcomes [21]. To improve outcomes, members must support, monitor and increase the community’s potential to learn by sharing and integrating each member’s knowledge, experiences and practices through interaction [16]. More specifically, given the importance of continuous learning in a knowledge economy in general [22, 23], and in healthcare in particular [24], there is a need to better understand what makes communities of practice effective at fostering learning.

A focus on what fosters learning in community of practices is important because it can also have a direct impact on resilience and patient safety. Resilience is “the capacity to adapt to challenges and changes at different system levels, to maintain high quality care” and it operates by way of “activities of learning and development through reflexive practice.” [25] – a key concept of communities of practice. Community of practice offer a context for adaptation, and adaptation is a key element of resilient interprofessional teams including when the focus is on improving patient safety. Communities of practice are also more democratic forums, with flat (or no) hierarchies, that focus the ownership of improvements and patient safety concerns to frontline workers [26].

This paper’s purpose is to study how members of a community of practice learn over time. Specifically, the goal of this paper is to assess how members of a community of practice perceive interactions among themselves and determine the extent to which these interactions predict self-assessed learning over time. Collaboration, psychological safety within the group and commitment to it are explored as longitudinal co-variants with learning. The following section presents the conceptual and logical arguments of self-assessed learning as a dependent variable.

### Dependent variable: self-assessed learning

“Learning is an active process within which learners construct new knowledge through interaction with their environment.” [27] This is true in communities of practice where learning is a central phenomenon taking place in contexts rich with interactions between people. Specifically, communities of practice provide context where interactions among members lead to learning from sharing of, and reflection on, experience [28]. Communities of practice espouse a reflective learning culture [29]. Learning occurs because members evolve from peripheral to core members of the community of practice in such a way that “knowledge inheres situatedly (sic) in practice and creeps into and occupies the community members when they work together.” [2].

Assessing one’s own learning is an essential part of learning [30] and a key component of being a skilled lifelong learner [31]. When healthcare professionals self-assess their learning, they reflect on the quality of their work and learning, and then make appropriate adjustments [32]. When members reflect on their group’s learning, they engage in a form of group reflexivity by gaining useful insights for improving the group’s learning trajectory. Learning self-assessment is therefore a key component of communities of practice.

Three predictors of self-assessed learning in a community of practice are examined, and conceptually and logically linked to learning. The first two predictors are perceptions of collaboration and psychological safety. The other is commitment towards the community of practice, understood as a disposition towards goal-directed behaviour.

### Independent variables

#### Interprofessional collaboration

“The main objective of interprofessional collaboration is to develop multi-perspective working where power, goals, decision-making, knowledge, and expertise are shared” [33] and the potential for developing interprofessional collaboration in healthcare is still great [34].

Communities of practice are based on the idea that knowledge is shared between individuals across professional, disciplinary or other boundaries [35]. Boundary crossing is the foundation on which concepts of inter-professional and trans-professional collaboration are based [36]. Collaboration is a “process whereby two or more social entities actively and reciprocally engage in joint activities aimed at achieving at least one shared goal.” [37] In general, group processes are important to coordinate and funnel resources, including knowledge, towards reaching desired outcomes [38]. Specifically, collaboration is related to healthcare team performance, especially when professionals with diverse expertise are required to interact [6, 39]. Interaction and collaboration to solve problems or create knowledge are fundamental to communities of practice [40]. When people interact in communities of practice to improve healthcare, they do so in order to bridge gaps in practices and thereby learn collaboratively by bringing different perspectives and worldviews to bear on problems [41]. Community members must make a conscious effort to meet and take time to conjure up innovative ways to solve problems — both despite and because of initial differences of perspective. Collaborative interactions imply that each party must communicate, coordinate and synchronize their thoughts and actions with those of others [42]. Hence, collaborative interactions occur when individuals exchange information and act on “who” does “what,” “why,” “how” and “when.” These deceptively simple questions are how each member’s practices come to be understood in the community. Without collaboration, there are no interactions or exchanges of practices, and consequently, according to community of practice theory, the potential for learning is thwarted. More importantly for our purposes, individuals’ self-assessment, awareness and judgment regarding collaboration occurring in the community of practice (i.e., perceptions of collaboration) signal whether and to what extent reciprocal reflection-in-action is occurring [43]. The extent to which members perceive they are collaborating tells them that they are participating in sharing their worldviews. How individual members perceive interpersonal coordination fosters impressions of relating to shared goals [44]. Accordingly, we hypothesize that individual members’ perception of collaboration in their community of practice is longitudinally and positively related to their self-assessed learning.

#### Psychological safety

Communities of practice theory tells us that the context in which members evolve must be mutual, which Wegner defines as trust in terms of individuals feeling “comfortable addressing real problems together and speaking truthfully.” [45] This is directly related to the construct of psychological safety. Psychological safety is a person’s sense (i.e., appreciation, perception) that others “will not embarrass, reject, or punish someone for speaking up.” [46] Edmondson and Lei [47] explain that psychological safety “facilitates the willing contribution of ideas and actions to a shared enterprise.” Individuals who perceive their environment as psychologically safe are more likely to manifest behaviours such as speaking up to those who occupy positions of authority at work, offering ideas, admitting mistakes, asking for help and providing feedback [47]. High psychological safety is key to effective and safe healthcare delivery, as well as organisational learning [48]. Psychological safety also serves as a basis for individual learning. When psychological safety is high individuals are prone to development, growth, change and behaviors such as asking questions, sharing thoughts and help-seeking [49]. Edmondson, Higgins, Singer and Weiner [50] invoke Kahn [51] to underscore that psychological safety “likely interacts with person-level characteristics to influence how individuals engage in their work …” [e.g., commitment, which we will address later] “… and thus their ability to alter their professional practice.” [50] The key idea is that when one person speaks up, more information is available to others, which increases the potential for learning. Consequently, we will test whether the personal sense of psychological safety that members feel in their community of practice is longitudinally and positively related to the self-assessed learning that occurs in it.

#### Commitment

Commitment is an integral feature of communities of practice [28]. For Etzioni [52], a community is characterized by people with affective ties who are committed to shared values, norms, meanings, history and identity [53]. Community of practice theory asserts that the passage from peripheral to core member of a community – which creates the learning – is determined by factors such as participation and commitment [54]. Commitment, which is considered a basic requirement along with values, trust and behavioural norms, mediates the relationship between the activities members practise in the community and the activity outcomes [55]. The need to commit is necessary because bridging the gaps in knowledge that bring the community together is an arduous process requiring persistence [56]. Individual members need to embrace this inherent difficulty because it drives “dynamic and informative interactions” that ultimately lead to individual learning [56]. In turn, persistence is a consequence of commitment [57]. Qualitative and case-based studies on communities of practice provide evidence for the importance of members’ commitment. For example, in a community of practice focused on changing professionals’ roles, individual commitment was related to how pertinent the new practices were in terms of avoiding duplication of effort [58]. Perception of commitment is also important. A simulation and laboratory study by Michael, Sebanz and Knoblich [44] found that perceptions of coordination in a joint effort affect individual members’ perceptions of the commitment of others. These authors hypothesize that the observability of interpersonal coordination facilitates inferences about the commitment of others. Hence, while individuals’ perceptions of various phenomena taking place at the group level are important, perceptions of commitment in communities of practice has, to our knowledge, not yet been examined either qualitatively or quantitatively. This is surprising given that individuals’ commitment is a key ingredient that holds members of a community of practice together [59]. Bate and Robert [60] suggest that one of the factors responsible for poor learning outcomes in communities of practice is the difficulty in maintaining the commitment of busy healthcare professionals. In this regard, we hypothesize that the commitment of members to their community of practice is longitudinally and positively related to their self-assessed learning in it.

To summarize, a focus on individual members’ commitment and their perceptions of what happens at the group level (i.e., collaboration, psychological safety) are important when considering learning in communities of practice. Interestingly, a review of 31 primary studies and two systematic surveys of healthcare communities of practice found that, despite the central role of learning in communities of practice, only seven studies discussed communities that had been explicitly established to foster individual learning [61] and only one study hints at improved learning [29]. In that descriptive study, average ratings of self-assessed learning after a training module on reflective learning events increased from 3.4 to 4.0 where 3 = sometimes and 4 = often, bearing in mind that no test to verify whether this difference was statistically significant was reported. Furthermore, the practical significance of a 0.6 difference is not clear at best. Building on the experience of the Heath Promotion Laboratories program [62, 63], our research aims to empirically investigate self-assessed learning in communities of practice by quantitatively and longitudinally exploring three potential predictors within them: commitment, collaboration and psychological safety.

## Methods

### Participants

Participants for this study are part of a larger initiative implemented in Québec (Canada) called the Health Promotion Laboratories program [62, 63] which was designed by a regional public health directorate as an innovative professional development intervention in health promotion [64, 65]. This program draws on the concept of communities of practice. A key learning strategy of the program involves supporting multidisciplinary teams of practitioners and managers in designing and implementing a health promotion intervention of their choice aimed at either developing new practices or improving existing ones. Communities of practice were recruited using a non-random sampling method. The Health and Social Services Centers were first approached by the Public Health Department (NB, 3rd author) to discuss their interest in participating. Then, managers and professionals in each establishment chose a theme for the lab work according to existing priorities. Once the theme was chosen, participants were mobilized on a voluntary basis by management. In total, six labs were composed of 83 managers and professionals from different disciplines and/or professions who voluntarily enrolled in the program. Table 1 summarises the mandate and what was implemented by each community of practice. These participants met for 3 h every two or 3 weeks for 18 to 36 months; their organizations allowed them to hold these meetings during scheduled work time.

**Table 1:** Community of practices’ mandate and health promotion interventions

	Mandate of the community of practice	Interventions implemented by the community of practice
A	To evaluate and prevent health risks among workers in the territory.	Supported the implementation of measures favorable to occupational health at the time of the start-up and relocation of companies in the territory.
B	Meet the needs of schools in the territory.	Supported the promotion of education by the parents of primary school students in the territory.
C	To intervene with families in matters of nutrition, vaccination, education, child behavior and family life.	Reserved childcare places for marginalized families; Established a breastfeeding promotion network; Developed a social network of immigrant mothers; Has set up a family/child consultation table in the territory.
D	To prevent disease and promote the health of pupils attending schools in its territory.	Developed activities to facilitate the smooth transition from primary school to secondary school.
E	Provide psychosocial services to vulnerable families in the territory with the aim of reducing the difficulties of social adaptation and its consequences.	Broke the isolation of community workers working in low-income housing by initiating joint projects; Has set up a collective kitchen in a low-rent dwelling.
F	Promote healthy lifestyle habits.	Created a community grocery store.

### Procedures

Four waves (i.e., four points in time) of measures of predictors and dependent variables were taken every 6 to 8 months in each of the six communities of practice. Questionnaires were completed individually and in silence prior to community of practice meetings. This study’s protocol was approved by the Université de Montréal ethics review board and carried out in accordance with the Declaration of Helsinki. Informed consent was obtained from all the participants by having them sign a consent form prior to providing data.

### Measures

Individuals’ perceptions of collaboration within their community of practice were measured using the Collaborative Work Questionnaire, with a 5-point frequency scale (1 = never or almost never; 5 = very often) [42]. An example from the 14-item questionnaire is “We exchange information on ‘who does what’” . A validation study confirmed a second order multi-level construct and internal consistency estimates above 0.77 [42]. Other empirical studies report alphas above 0.80 [66, 67].

Individuals’ perceptions of psychological safety within their community of practice were measured using Edmondson’s 7-item instrument [46]. A 5-point agreement scale (1 = completely disagree; 5 = completely agree) was used with items such as “It is safe to take a risk on this team”. Applying Edmondson’s initial validation using factor analysis shows a clear unidimensional construct and adequate internal consistency (i.e., α = 0.92).

Individuals’ perceptions of commitment within their community of practice were evaluated using five items from an instrument measuring project commitment [68] and by changing “project” for “health promotion project,” as in “This health promotion project has our strong commitment”. The same agree-disagree response format was used. Original factor analyses reveal a single construct measure, and internal consistency was α = 0.75 [68].

For the dependent variable, we adapted six items pertaining to individuals’ perceptions of learning and development from an instrument developed by García-Ramírez, Paloma, Suarez-Balcazar and Balcazar [69]. Items such as “Participants in this Lab aimed at continuous improvement and feedback” and “The Lab helped its members identify and strengthen their skills” were measured using a 5-point agreement scale (1 = completely disagree; 5 = completely agree). A systematic review and integrated model by Foster-Fishman, Berkowitz, Lounsbury, Jacobson and Allen [70] for developing core competencies and processes related to collaborative capacity supports the statements used by García-Ramírez, Paloma, Suarez-Balcazar and Balcazar. While these authors did not provide internal consistency estimates, our sample (i.e., Table 2) shows Cronbach’s alphas ranging from 0.60 to 0.83 across our four waves.

**Table 2:** Descriptive statistics and reliability estimates

	N	*M* ^a^	*SD*	Internal consistency ^b^
Wave 1				
Collaboration	55	3.644	0.703	0.942
Psychological safety	55	3.854	0.555	0.630
Commitment	55	3.724	0.624	0.859
Learning and dev.	55	3.445	0.542	0.827
Wave 2				
Collaboration	46	3.754	0.602	0.910
Psychological safety	46	4.043	0.476	0.612
Commitment	46	4.017	0.540	0.717
Learning and dev.	46	3.760	0.499	0.794
Wave 3				
Collaboration	51	3.633	0.522	0.888
Psychological safety	51	3.835	0.458	0.609
Commitment	51	3.969	0.543	0.842
Learning and dev.	51	3.627	0.369	0.604
Wave 4				
Collaboration	49	3.815	0.620	0.942
Psychological safety	49	4.003	0.457	0.587
Commitment	49	4.002	0.572	0.861
Learning and dev.	49	3.707	0.497	0.753

Notes

^a^ All response formats varied from 1 to 5.

^b^ Cronbach’s alpha (α).

### Analyses

We used software package IBM SPSS and generalized estimating equations (GEEs) to test whether perceptions of collaboration, psychological safety and commitment to the group are longitudinally and positively related to self-assessed learning. GEEs are particularly suited for understanding how a dependent variable changes over time as a function of independent variables, especially when data are expected to be correlated [71]. Moreover, this technique is robust with respect to missing data because not all time points need to have all measures (i.e., all available pairs of data are used in estimating the working correlation parameters and includes them in the estimation of the variances [72]) making imputation less necessary [73–76]. GEEs produce accurate standard errors which benefits confidence intervals [74]. In addition, GEEs are relevant for studies in organizations and work settings because they can account for within-subject correlations and are robust with respect to variables that are not necessarily distributed normally [72, 77].

## Results

A total of 83 health and social service professionals from six organizational communities of practice in the province of Quebec agreed to take part in this study. Our data show that 21 participants (25.3%) took part in one wave of measures, 21 (25.3%) in two, 16 (19.3%) in three, and 22 (23.5%) in all waves, while 3 (3.6%) did not provide measures in any waves. Participants’ mean age was 47.2 years old (SD = 8.9). Fifteen (18.5%) were men, 66 (79.5%) were women; 2 participants had missing gender data. Twenty-seven (33.8%) were social workers, 22 (27.5%) were nurses and 34 (38.7%) were in other occupations such as nutritionist or manager; 3 participants did not provide their occupation. These participants had spent a mean of 11.5 years (SD = 7.9) in their organization and a mean of 8.2 years (SD = 7.6) in their current position. Table 2 shows our sample’s descriptive data for dependent and independent variables including internal consistency. Cronbach’s alphas are acceptable across all waves except for psychological safety and for one point in time for self-assessed learning which could have been higher.

Table 3 shows two GEE models. The first is the naive model similar to what would have been obtained with ordinary least squares regression. It is used as a baseline to compare more adequate models. The second model uses an autoregressive working correlation matrix to estimate parameters with a gamma distribution for the dependent variable. It emerged as superior to the naive model as indicated by much smaller quasi-likelihood under independence model criterion (QIC) and corrected quasi-likelihood under independence model criterion (QICC) fit indices. Overall, the second model shows that all three independent variables are, as predicted, positively and longitudinally related to self-assessed learning.

**Table 3:** Generalized Estimating Equation analyses for collaboration, psychological safety and commitment interacting with community of practice and for time as within-subject factor in predicting learning (*O*^a^ = 201)

Parameter	β	SE	LLCI^b^	ULCI^c^	Wald	Exp(β)	LLCI^b^	ULCI^c^
Normal distribution, Identity link, Independent model QIC^d^ = 45.612; QICC^e^ = 41.030								
Intercept	1.362	0.2668	0.839	1.885	26.069***	3.905	2.315	6.587
Collaboration	0.144	0.0569	0.032	0.255	6.408*	1.155	1.033	1.291
Psychological safety	0.087	0.0658	−0.042	0.215	1.730	1.090	0.958	1.240
Commitment	0.355	0.0581	0.242	0.469	37.469***	1.427	1.273	1.599
Gamma distribution, Identity link, Autoregressive model QIC^d^ = 14.070; QICC^e^ = 10.548								
Intercept	1.480	0.2604	0.970	1.990	32.308***	4.393	2.637	7.318
Collaboration	0.114	0.0564	0.004	0.225	4.115*	1.121	1.004	1.252
Psychological safety	0.138	0.0655	0.009	0.266	4.416*	1.148	1.009	1.305
Commitment	0.298	0.0584	0.183	0.412	25.951***	1.347	1.201	1.510

Notes

^a^ Observations: all non-missing observations (i.e., 60.5%) from 83 individuals measured 4 times.

^b^ LLCI: 95% lower limit confidence interval.

^c^ ULCI: 95% upper limit confidence interval.

^d^ QIC: quasi-likelihood under independence model criterion; the smaller the better.

^e^ QICC: corrected quasi-likelihood under independence model criterion; the smaller the better.

* *p* ≤ .05; ** *p* ≤ .01; *** *p* ≤ .005; all Wald statistics calculated with 1 df.

Specifically, collaboration is a positive predictor of self-assessed learning (β = 0.114, Wald = 4.115, *p* ≤ 0.05). This means that perceptions of collaboration among community of practice members is associated with self-assessed learning over time. Second, a member’s personal sense of psychological safety in the community of practice is a predictor that is longitudinally associated with perception of learning (β = 0.138, Wald = 4.416, p ≤ 0.05). A sense of safety in the group and the ability to speak up correlate well with individuals’ self-appreciation of learning over time. Finally, commitment to the community of practice is also positively predictive of self-assessed learning (β = 0.298, Wald = 25.951, *p* ≤ 0.001). This means that over time the extent to which members of the community commit to the community’s purpose is associated to individuals’ perception of learning.

## Discussion

### Contribution to research

Our aim was to quantitatively understand key individual perceptions that take place in a community of practice over time. Our results show how an individual member’s feelings about collaboration, psychological safety and commitment to the group are longitudinally and positively associated with self-perceptions of learning in their community of practice. As such this study makes four contributions to research.

First, collaboration is positively related to self-assessed learning. Collaboration fosters interactions and exchange of practices, and, in terms of community of practice theory, therefore, fosters learning. Specifically, the more members of a community of practice collaborate, the more they will learn from each other over time. Collaborative interactions occur when individuals exchange information and act on “who” does “what,” “why,” “how” and “when.” Such interactions help bring different perspectives and worldviews to bear on problems [41], while requiring that each community member communicate, coordinate and synchronize their thoughts and actions with those of others [42]. Collaboration and learning have close theoretical connections and our results hint at future research in that direction. For example, future research should focus on distinguishing between individual-level learning (i.e., learning in the context of a community of practice) and group-level learning (i.e., a change in a community of practice’s repertoire of behaviours) [78] as a function of collaboration intensity. This is logically derived from researchers who suggest that collaboration is at its height when cases are complex, when there is interdependence and interdisciplinarity between professionals, and when professionals share practices and engage in shared decision-making [79]. Hence, future research should investigate whether there is a point in time where learning in a community of practice goes from individual learning (where collaboration is at its minimum) to learning where collaboration is at its most intense.

Second, Edmondson, Higgins, Singer and Weiner [50] recently noted the paucity of research on psychological safety and adult learning. Recent work suggests that it is still unclear whether psychological safety acts as a direct enhancer of learning or as a remover of barriers to learning [50, 80]. Our longitudinal results suggest that psychological safety may enhance how people feel they learn over time. Future research should attempt to replicate our study and examine whether perceptions of psychological safety are longitudinally associated with learning as a function of community of practice members’ status. Indeed, even if communities of practice are spaces characterized by respect for the diversity of views and minority opinions [56], Edmondson, Higgins, Singer and Weiner [50] found that the status and hierarchical issues at play in healthcare result in stronger perceptions of psychological safety for high-status individuals. The suggestion that lower-status community members might feel less inclined to share freely and the impact of this feeling on perceptions of learning deserves further research.

Third, we expected commitment to the group to stand out due to its importance in community of practice theory [81]. Commitment has to do with an individual member’s attachment to, and identification with, a given entity such as an organization or a group [82]. This is also how it is understood in community of practice theory [45]. However, it is possible to go further. Allen and Meyer explain that there are three forms of commitment: affective (i.e., engaging because of an emotional attachment), normative (i.e., engaging because of a perceived moral obligation), and continuance (i.e., engaging because it would be too costly not to do so). While we found evidence for commitment in data stemming from the research program this article is a component of [63], with hindsight we feel that contextual variables may have triggered forms of commitment we were not equipped to measure. The meta-analysis by Meyer, Stanley, Herscovitch, and Topolnytsky [83] is revealing in this regard. They found that high affective commitment is associated with environments where the work concerned is well defined (i.e., low role ambiguity). They show that high continuance commitment is associated with environments where actors are asked to play incompatible roles (i.e., high role conflict). Finally, the meta-analysis revealed that high organizational support is associated with high normative commitment. Although Meyer, Stanley, Herscovitch, and Topolnytsky’s meta-analysis is not specific to communities of practice, this tri-dimensional view of commitment is fertile ground for better understanding how commitment is linked to learning in communities of practice.

The final contribution this study makes has to do with methodology. Given that most studies on communities of practice are qualitative and cross-sectional, our study provides longitudinal quantitative input to extant literature.

### Contribution to practice

Our take-away message is that communities of practice can apply these findings by making collaboration, psychological safety, commitment and learning regular topics of reflexive discussion so that group norms on teamwork, openness towards others’ views, and commitment to the community of practice can be understood, monitored, and integrated. Discussing learning is important because “it is often hard for workers to recognize that any learning is taking place while they are working.” [23] More specifically, leaders of communities of practice in work settings might wish to foster two complementary forms of learning behaviours in their community: collective insight and change production. Edmondson [4] explains that fostering collective insight involves ensuring information is shared and feedback provided, that problems or errors are discussed, and that experiments are undertaken to increase awareness. She also explains that bringing about change requires focus on effective decision-making and planning as well as working on improving performance and knowledge transfer. Drawing on the literature to date on team reflexivity and team adaptation [84, 85], regular reflexivity discussions based on answers to such questions as “what are our goals, did they change, and if so, why?” or “are we actually acquiring knowledge from discussions with one another?” or “what group rules can we develop and monitor that would ensure we all feel respected?” are likely to foster psychological safety. In addition to reflexive discussions based on these questions, certain topics should also be addressed, such as reporting errors. Learning from one’s own mistakes is an important concept for professionals [43, 86], and by extension, learning from others’ mistakes in communities of practice is also an important element for developing individual members’ competencies. Interestingly, reporting errors is positively associated with psychological safety [87].

### Limitations

Our study has limitations that need to be underscored. First, while effective learners gain insights from reflecting on their achievements [43, 86], individuals’ assessments of their own learning can be considered potentially “inaccurate,” even if health professions emphasize the ability to self-assess [88]. Reviews suggest that there are many valid reasons why self- and other-ratings could differ, and that there is generally more agreement than disagreement in studies of self- and other-ratings [89]. Nevertheless, future studies in work settings should add an assessment from an instructor or immediate superior, preferably not questionnaire-based. Second, GEEs are imperfect for taking into consideration the impact of clusters (i.e., when individuals are nested in groups such as in our case with communities of practice) when the number of clusters is small [74]. A more sophisticated multi-level analytical strategy that examines how individual-level phenomena emerge over time to impact group-level outcomes would have been better. Unfortunately, we did not have enough statistical power to conduct such analysis. Furthermore, emergence – when interactions between individuals emerge over time as a group-level phenomenon – is not a mature field at this time because it lacks proper quantitative analytical tools for naturalistic data [8]. Third, although questionnaires made it possible to gather and analyse data quantitatively over time, their use as a single method can be criticized. The integration of qualitative data collection and analysis to a quantitative approach would have been more reveling of processes that occurred over time [90]. Finally it is worth noting that some internal consistency estimates are lower than expected (Table 2). Results pertaining to psychological safety should be regarded as tentative until replicated.

## Conclusion

One common theme to the research and practice issues we raised concerns longitudinal phenomena such as perceptions of collaboration, commitment, psychological safety and learning in communities of practice. In terms of conceptual development and theory, our results suggest that collaboration stimulates interactions that are crucial for learning in a community of practice, that psychological safety is an enhancer of learning rather than a remover of barriers to learning, and that commitment to the community of practice is strongly associated with learning. In practical terms, our study suggests that communities of practice can improve their learning by engaging in reflexive discussions about collaboration, commitment, and psychological safety at an early stage in their activities.

## Data Availability

The datasets generated and/or analysed during the current study are not publicly available because such information was not part of the consent form participants signed.

## Abbreviations

GEE: Generalized estimating equation
QIC: Quasi-likelihood under independence model criterion
QICC: Corrected quasi-likelihood under independence model criterion
